# Stilbene glycosides are natural product inhibitors of FGF-2-induced angiogenesis

**DOI:** 10.1186/1471-2121-10-30

**Published:** 2009-04-23

**Authors:** Sajjad Hussain, Mark Slevin, Nessar Ahmed, David West, Muhammad Iqbal Choudhary, Humera Naz, John Gaffney

**Affiliations:** 1 Department of Paediatrics and Adolescent Medicine, Mayo Clinic, Rochester, MN 55902, USA; 2 School of Chemistry, Biology and Health Sciences, Manchester Metropolitan University, Chester St, Manchester, M1 5GD, UK; 3 School of Biological Sciences, University of Liverpool, Liverpool, L69 7ZB, UK; 4 HEJ Research Institute of Chemistry, International Centre for Biological and Chemical Sciences, University of Karachi, Karachi 75720, Pakistan

## Abstract

**Background:**

Angiogenesis, the growth of new blood vessels from the pre-existing vasculature is associated with pathological processes, in particular tumour development, and is a target for the development of new therapies. We have investigated the anti-angiogenic potential of two naturally occurring stilbene glycosides (compounds **1 **and **2**) isolated from the medicinal plant *Boswellia papyriferai *using large and smallvessel-derived endothelial cells. Compound **1 **(trans-4',5'-dihydroxy-3-methoxystilbene-5-O-{α-L-rhamnopyranosyl-(1→2)-[α-L-rhamnopyranosyl-(1→6)}-β-D-glucopyranoside was the more hydrophilic and inhibited FGF-2-induced proliferation, wound healing, invasion in Matrigel, tube formation and angiogenesis in large and small vessel-derived endothelial cells and also in the chick chorioallantoic membrane assay. Using a binding assay we were able to show compound **1 **reduced binding of FGF-2 to fibroblast growth factor receptors-1 and -2. In all cases the concentration of compound **1 **which caused 50% inhibition (IC_50_) was determined. The effect of compound **1 **on EGF and VEGF-induced proliferation was also investigated.

**Results:**

Compound **1 **inhibited all stages of FGF-2 induced angiogenesis with IC_50 _values in the range 5.8 ± 0.18 – 48.90 ± 0.40 μM but did not inhibit EGF or VEGF-induced angiogenesis. It also inhibited FGF-2 binding to FGF receptor-1 and -2 with IC_50 _values of 5.37 ± 1.04 and 9.32 ± 0.082 μM respectively and with concommotant down-regulation of phosphorylated-ERK-1/-2 expression. Compound **2 **was an ineffective inhibitor of angiogenesis despite its structural homology to compound **1**.

**Conclusion:**

Compound **1 **inhibited FGF-2 induced angiogenesis by binding to its cognate receptors and is an addition to the small number of natural product inhibitors of angiogenesis

## Background

Angiogenesis, the formation of new blood vessels from the pre-existing vasculature, is a closely regulated sequence of events beginning with the degradation of the basement membrane by activated endothelial cells (ECs). These then migrate and proliferate, form endothelial sprouts and develop capillary tubes and a new basement membrane. The key events of angiogenesis therefore involve EC proliferation, migration, tube formation and differentiation into capillaries [1]. Angiogenesis is associated with normal physiological (wound healing, endometrial cycle and embryonic development) and pathological processes (tumour growth, rheumatoid arthritis, diabetic retinopathy, and brain and cardiac infarctions) [2-4].

Angiogenesis is regulated by a balance between endogenous, soluble pro-angiogenic factors (including vascular endothelial cell growth factor (VEGF) [5], fibroblast growth factor-2 (FGF-2) [6], epidermal growth factor (EGF) and angiopoietins, and anti-angiogenic factors (including transforming growth factor-β, endostatin and thrombospondin) [7-9]. Growth factors exert their effect through binding to their cognate receptor; for example the kinase insert domain-containing receptor (VEGF) and Tie-2 receptors (angiopoietins) [10]. FGFs exert their effect by binding to high affinity FGF-receptors (FGF-R) on the cell surface. *In vitro*, ECs express FGFR-1 and in some cases FGFR-2 but not FGFR-3 or -4 [11].

Because de-regulated angiogenesis is associated with disease progression, especially tumour development, inhibition of neo-vessel growth has become a target in drug development. Natural compounds from medicinal plants display diverse pharmacological activities and have advantages over synthetic drugs, such as smoother action, better tolerance and fewer allergic reactions [12]. For example anti-angiogenic plant derived natural products such as genistein [13], isoliquitrin [14], ginsenoside[15] and torilin [16] have potent effects on EC proliferation or tube formation.

**Figure 1:**
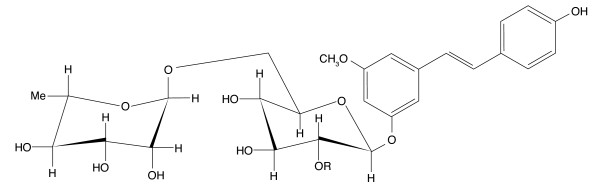
**The structures of the stilbene glycosides used in the study**. Compound **1 **(R = -L-rhamnose) and **2 **(R = H).

Stilbene glycosides are natural products isolated from the medicinal plant *Euphobia chiradenia *and in preliminary screening were shown to be PLA_2 _inhibitors, have anti-inflammatory properties and inhibit wound healing although the mechanism of action was not investigated [17]. Based on these results we speculated that stilbene glycosides may be anti-angiogenic and tested the efficacy of two of these compounds, trans-4',5'-dihydroxy-3-methoxystilbene-5-O-{α-L-rhamnopyranosyl-(1→2)- [α-L-rhamnopyranosyl-(1→6)}-β-D-glucopyranoside (compound **1**) and trans-4',5'-dihydroxy-3-methoxystilbene-5-O-[α-L-rhamnopyranosyl-(1→6)]-β-D-glucopyranoside (compound **2**) (Figure 1; see methods) against large and small vessel-derived EC in a range of *in vitro *and *in vivo *angiogenic assays.

## Results

### Toxicity

**Figure 2:**
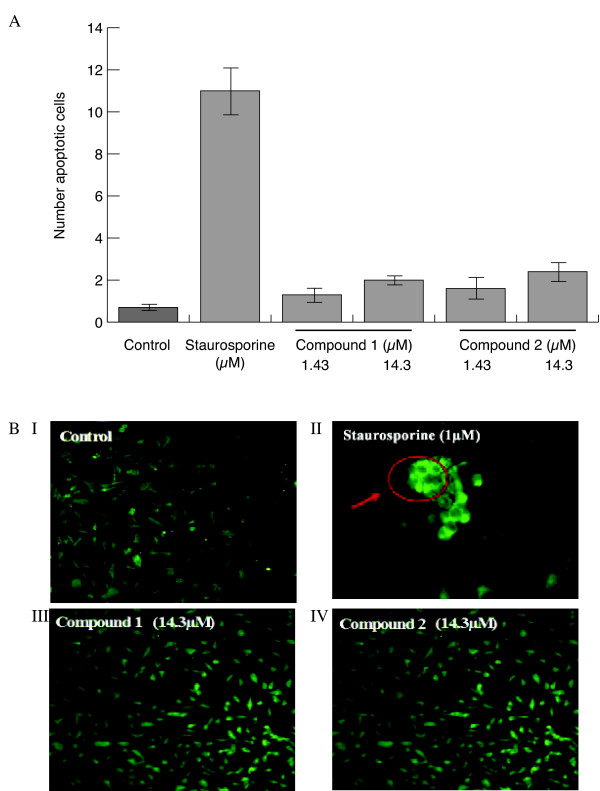
**The effect of compounds 1 and 2 on BAEC viability**. The cytotoxic effect was determined using **(A) **The MTT assay; cells (7.5 × 10^3^) were incubated with the test compounds or with staurosporine (1.4 μM) an inducer of active caspase-3 and of apoptosis for 72 h and MTT added. The absorbance was read at 570 nm. **(B)** Active-caspase-3 apoptosis assay: cells (4.0 × 10^4^/ml) were incubated with the test compounds or with staurosporine (1.0 μM, 24 h) and stained with anti-active caspase-3 as described below. Experiments were performed in triplicate. Representative immunofluorescence photomicrographs for BAEC were taken as described below. A group of apoptotic cells are highlighted in II.

Compounds **1 **and **2 **had no significant cytotoxic effect on bovine aortic endothelial cells (BAEC) and human dermal microvascular endothelial cells (HDMEC) over the concentration range used whereas staurosporine (an inducer of active caspase-3 and a positive control) showed significant cytotoxicity. Representative data for BAEC are shown in Figure 2.

### The effect of compounds 1 and 2 on growth factor-induced proliferation

**Figure 3:**
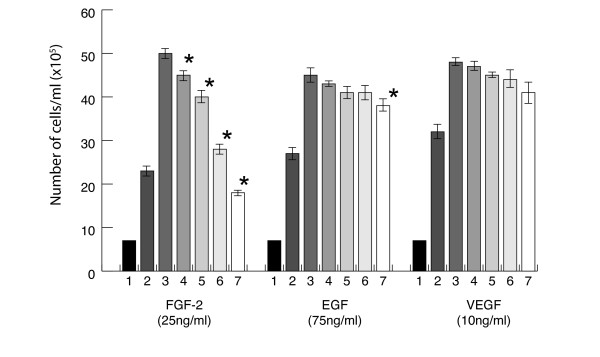
**The effect of compound 1 on growth factor induced BAEC proliferation**. Cells were seeded in a 6-well plate in the numbers shown and the effect of compound **1 **on growth factor-induced proliferation (25–75 ng/ml) was determined as described below. Columns 1 seeded cells; 2 DMSO alone; 3 growth factor alone; 4–7 compound **1 **at concentrations of 1.43, 14.3, 28.6 and 71.5 μM. Values which differ significantly (p < 0.05) from growth factor alone are indicated by *. Results are the mean of three experiments.

Compounds **1 **and **2 **at concentrations of 1.4–71.5 μM had no significant effect on BAEC and HDMEC growth in the absence of growth factors (Figure 3).

Over a range of concentrations compound **1 **inhibited FGF-2-induced BAEC and HDMEC proliferation in a dose dependent manner with IC_50 _values of 48.90 ± 0.40 and 42.0 ± 0.93 μM respectively (p < 0.05 in both cases). Compound **2 **was a less effective inhibitor and inhibited HDMEC (IC_50 _of 101.0 ± 0.50 μM; p < 0.05) but had no activity against large vessel BAEC (Table 1). The compounds had little effect on vascular endothelial growth factor (VEGF) or epidermal growth factor (EGF) stimulated proliferation (Figure 3).

**Table 1:** A summary of the anti-angiogenic properties of stilbene glycosides.

**Assay**	**Compound 1**		**Compound 2**	
	BAEC	HDMEC	BAEC	HDMEC
Proliferation in 15% FBS	NS	NS	NS	NS
EGF-induced proliferation	NS	NS	NS	NS
VEGF-induced proliferation	NS	NS	NS	NS
FGF-2-induced proliferation	48.90 ± 0.40	42.0 ± 0.93	NS	101.0 ± 0.50
FGF-2-induced migration	41.8 ± 0.95	ND	NS	ND
FGF-2-induced chemotaxis	30.05 ± 0.85	21.50 ± 0.6	800.0 ± 2.78	953.1 ± 3.50
FGF-2-induced tube formation	18.20 ± 0.65	12.4 ± 0.85	NS	NS
FGF-2 invasion in matrigel	11.12 ± 0.28	5.8 ± 0.18	18.90 ± 0.50	32.46 ± 0.95
Tubular regression	40.0 ± 0.2	ND	37.42 ± 0.63	ND
Effect on binding to FGFR-1	5.37 ± 1.04		NS	
Effect on binding to FGFR-2	9.32 ± 0.082		NS	
*In vivo *CAM assay	100% inhibition		90% inhibition	

NS = Not significant, overall inhibition is less than 50% and therefore IC_50 _values cannot be calculated. ND = not determined

Figures are the IC_50 _values ± SD (μM) and are the mean of at least three determinations.

### The effect of compounds 1 and 2 on endothelial cell migration during wound healing

**Figure 4:**
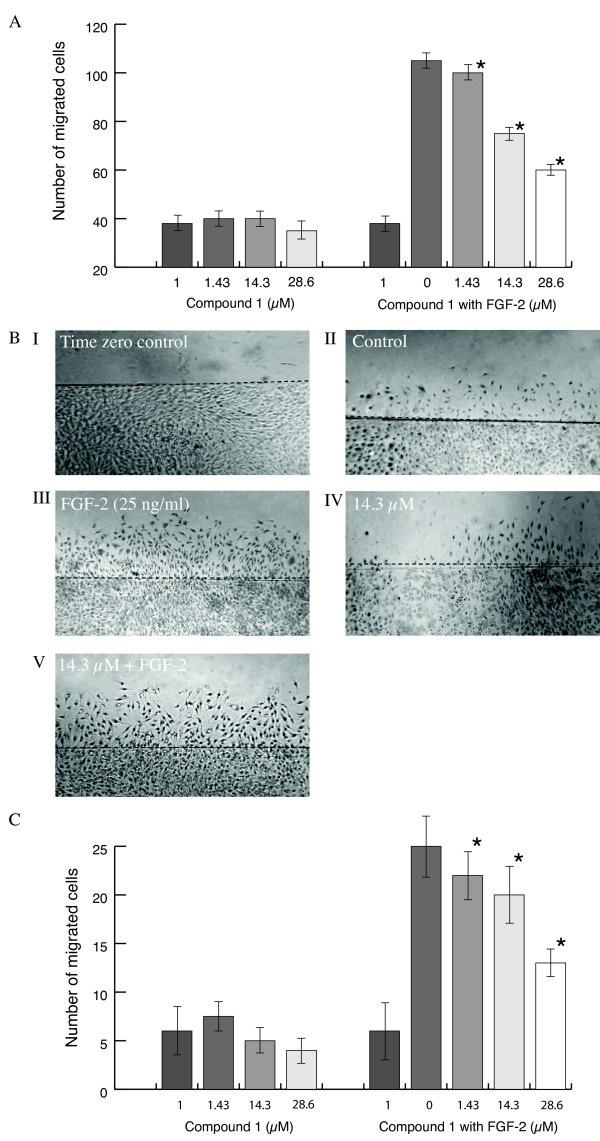
**Effect of stilbene glycosides on FGF-2 induced BAEC migration**. **(A) **A cell monolayer was wounded as described below and recovery was assessed in the presence of increasing concentration of the test compounds. Results are shown for compound **1**. Values significantly different from FGF-2 alone (p < 0.05) are shown by *. **(B)** Representative photomicrographs (original magnification × 20) show the effect of compound **1 **on BAEC migration. DMSO was used as the control. **(C)** The effect compound **1 **on FGF-2-induced cell migration in a three dimensional Boyden chamber assay. Cells were added to the upper chamber and the compound at a range of concentrations, with and without FGF-2 (25 ng/ml) added to the lower chamber (see below for method). The total number of migrated cells to the lower chamber was counted as described below. Column 1, control. The results are the mean of three experiments and values significantly different from FGF-2 alone (p < 0.05) are shown by *.

Two- and three-dimensional cell migration assays were used to determine whether compounds **1 **and **2 **had an effect on EC cell migration. In a wound healing model, cell monolayers were wounded and exposed to compounds **1 **or **2 **with and without FGF-2. FGF-2 at a final concentration of 25 ng/ml induced significant migration into the denuded area (p = 0.001) and this was inhibited by compound **1 **with an IC_50 _value of 41.80 ± 0.95 μM (p < 0.05; Figure 4A). Compound **2 **was ineffective. Representative photomicrographs are shown (Figure 4B).

The chemotactic effect of compounds **1 **and **2 **was measured in the three-dimensional Boyden chamber assay. BAEC and HDMEC stimulated by FGF-2 (25 ng/ml) showed a significant increase in migration (p < 0.05 in both cases). Compound **1 **inhibited migration of both cell types in a dose-dependent manner with IC_50 _values of 30.05 ± 0.85 and 21.50 ± 0.6 μM respectively (p < 0.05; Figure 4C). Compound **2 **was ineffective with IC_50 _values in excess of 800 μM.

### The effect of compounds 1 and 2 on endothelial tube formation

**Figure 5:**
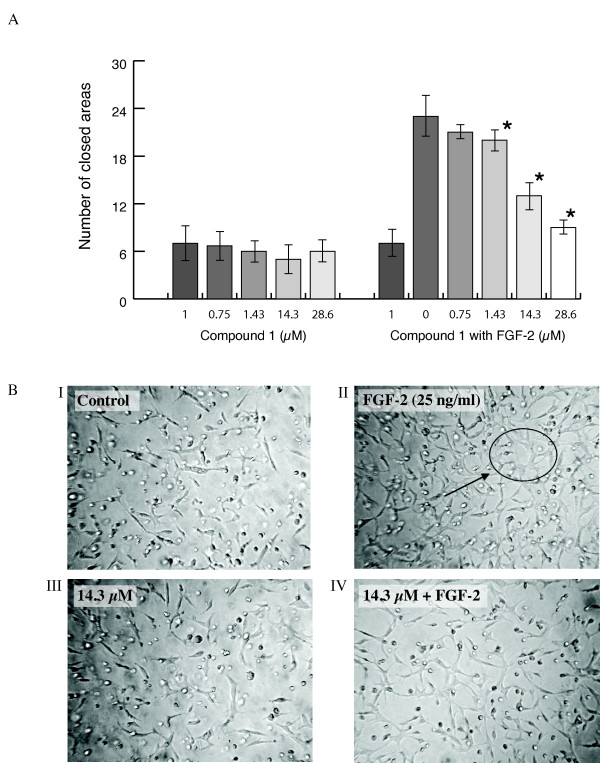
**The effect of compound 1 on BAEC tube formation in Matrigel with and without FGF-2**. **(A)** Compound **1 **inhibited FGF-2 (25 ng/ml) induced BAEC differentiation into capillary like structures: column1, control. In the absence of FGF-2 there was no inhibitory effect on tube formation. Values significantly different from FGF-2 alone (p < 0.05) are shown by *. Results are the mean of three experiments **(B)** Representative photomicrographs (original magnification × 20) show tube formation by BAEC. In the presence of FGF-2 a closed tubular network was evident (an example is shown in II) and this was abolished by compound **1 **(shown in IV). In the control (I0 and with compound **1 **alone (III) there was no evidence of tube formation.

In the presence of FGF-2 (25 ng/ml) there was an increase in BAEC and HDMEC differentiation into capillary-like structures (approximately 4.0 fold; p = 0.007 in each case: Figure 5A). This process was inhibited by compound **1 **in a dose dependent manner with IC_50 _values of 11.12 ± 0.28 and 5.8 ± 0.18 μM (p < 0.05 in both cases) for BAEC and HDMEC respectively. Figure 5 shows representative results for BAEC. Compound **2 **had no inhibitory activity.

**Figure 6:**
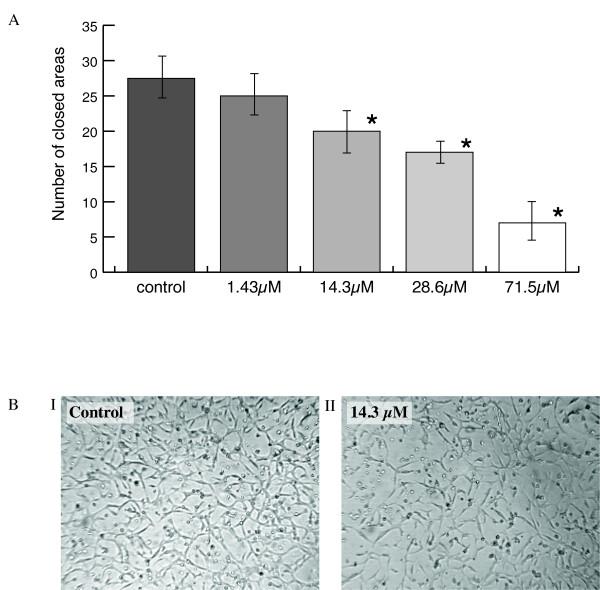
**(A) The effect of compound 1 on the regression of established BAEC tubular networks**. Cells were treated with FGF-2 on matrigel until an established tubular network was visible. The test compounds were added in serum and after 24 h the number of closed areas was counted as described below. The results are the mean of three experiments and values differing from the control are shown by *. (B) Representative photomicrographs of tubular structures shown for FGF-2 alone (control; panel I) and in the presence of compound **1 **(panel II) show tubular regression in the presence of the test compound (original magnification × 20).

The effect of compounds **1 **and **2 **on the regression of established tubular networks formed from BAEC was also investigated. Compound **1 **had an IC_50 _value of 40.0 ± 0.2 and compound **2 **of 37.42 ± 0.63 μM respectively (p < 0.05 in both cases; Figure 6 shows representative results).

### The effect of compounds 1 and 2 on endothelial invasion in Matrigel

**Figure 7:**
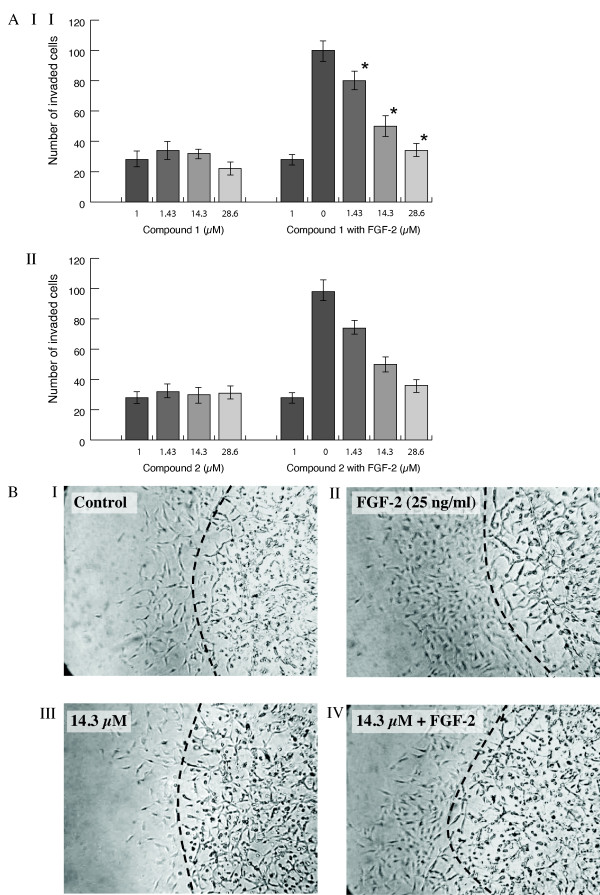
**The effect of compounds 1 (AI) and 2 (AII) on BAEC invasion into a Matrigel layer was studied using the chemoinvasion assay described below**. Cells (1.7 × 10^4^) were added to the Matrigel coated upper Boyden chamber and the compounds with or without FGF-2 added to the lower chamber. Cell invasion was measured after 24 h and assessed as described below. Values significantly different (p < 0.05) from FGF-2 alone are shown by *. Results are the mean of three experiments. **(B) **Representative photomicrographs are shown for the effect of compound **1 **on BAEC (original magnification × 20). Increased migration was seen with FGF-2 (25 ng/ml; panel II) and this was reduced in the presence of compound **1 **(panel IV).

The effect of the test compounds on cell invasion was investigated using a Transwell Boyden chamber system coated with reconstituted growth factor-depleted Matrigel. BAEC and HDMEC treated with FGF-2 showed a 3.2 and 3.0 fold increase in migration into a second layer of Matrigel respectively (p = 0.002). This process was inhibited by compound **1 **with IC_50 _values of 11.12 ± 0.28 and 5.8 ± 0.18 μM (p < 0.05 in both cases; Figure 7A) and compound **2 **with IC_50 _values of 18.90 ± 0.50 and 32.46 ± 0.95 μM (p < 0.05) for BAEC and HDMEC respectively. Representative photomicrographs for the effect of compound **1 **are shown (Figure 7B).

### Binding studies with FGF-2 receptors

**Figure 8:**
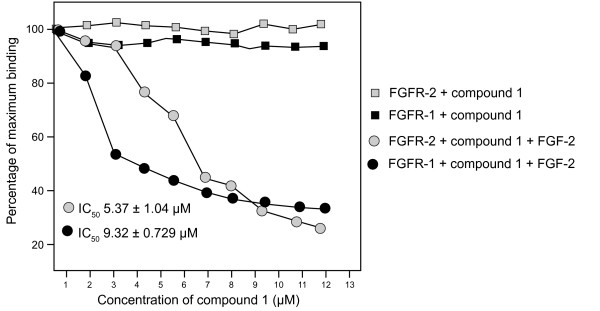
**The effect of increasing concentrations of compound 1 on FGF-2 binding to FGFR-1 and -2**. The soluble receptor was coated onto the wells of a 96-well plate and blocked with 1% BSA. The test compounds and growth factor were premixed for 2 h and added to the plate. After 2 h the plate was washed and incubated with antibodies to the growth factor, then peroxidase-conjugated IgG for 45 min. Peroxidase substrate was added and the absorbance read at 405 nm (se below for detail).

The anti-angiogenic effect of compounds **1 **and **2 **may occur by either competing with FGF-2 for its receptors or by altering receptor binding. If compound **1 **and **2 **were added with FGF-2 to FGFR-1 or FGFR-2 no significant inhibitory effect was observed. However, if the compounds were pre-incubated with FGF-2 a significant reduction of FGF-2 binding for compound **1 **(IC_50 _= 9.32 ± 0.729 μM for FGFR-1 and 5.37 ± 1.04 μM for FGFR-2) was observed (Figure 8). Compound **2 **had no inhibitory effect and neither compounds affected binding to the VEGF receptor (results not shown).

### The inhibition of ERK1/2 phosphorylation by compounds 1 and 2

**Figure 9:**
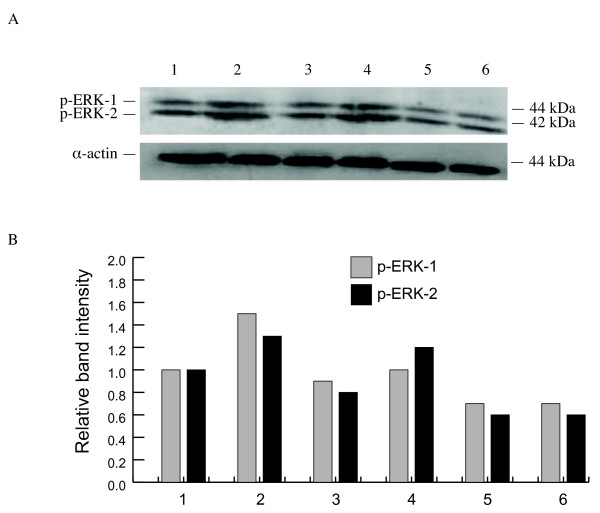
**(A) Western blots of p-ERK-1 and -2 were performed on BAEC treated with compound 1 as described below**. **(B) **The bar charts show relative protein expression compared to the control given an arbitrary value of 1.0. All experiments were performed at least twice and a representative example is shown. Lane 1 control, lane 2 FGF-2 (25 ng/ml), lane 3 compound **1 **(1.43 μM), lane 4 compound **1 **(14.3 μM) + FGF-2, lane 5 compound **1 **(28.6 μM), lane 6 compound **1 **(28.6 μM) + FGF-2.

FGF-2 induced cell proliferation, migration and differentiation is mediated through receptor binding and associated intracellular signal pathways involving ERK1/2 [18]. FGF-2 addition caused a 50% increase in ERK1/2 phosphorylation compared to the control. This was inhibited by compound **1 **in a dose-dependent manner (Figure 9A and 9B). Compound **2 **was ineffective.

### Inhibition of angiogenesis in the chick chorioallantoic membrane (CAM) assay

**Figure 10:**
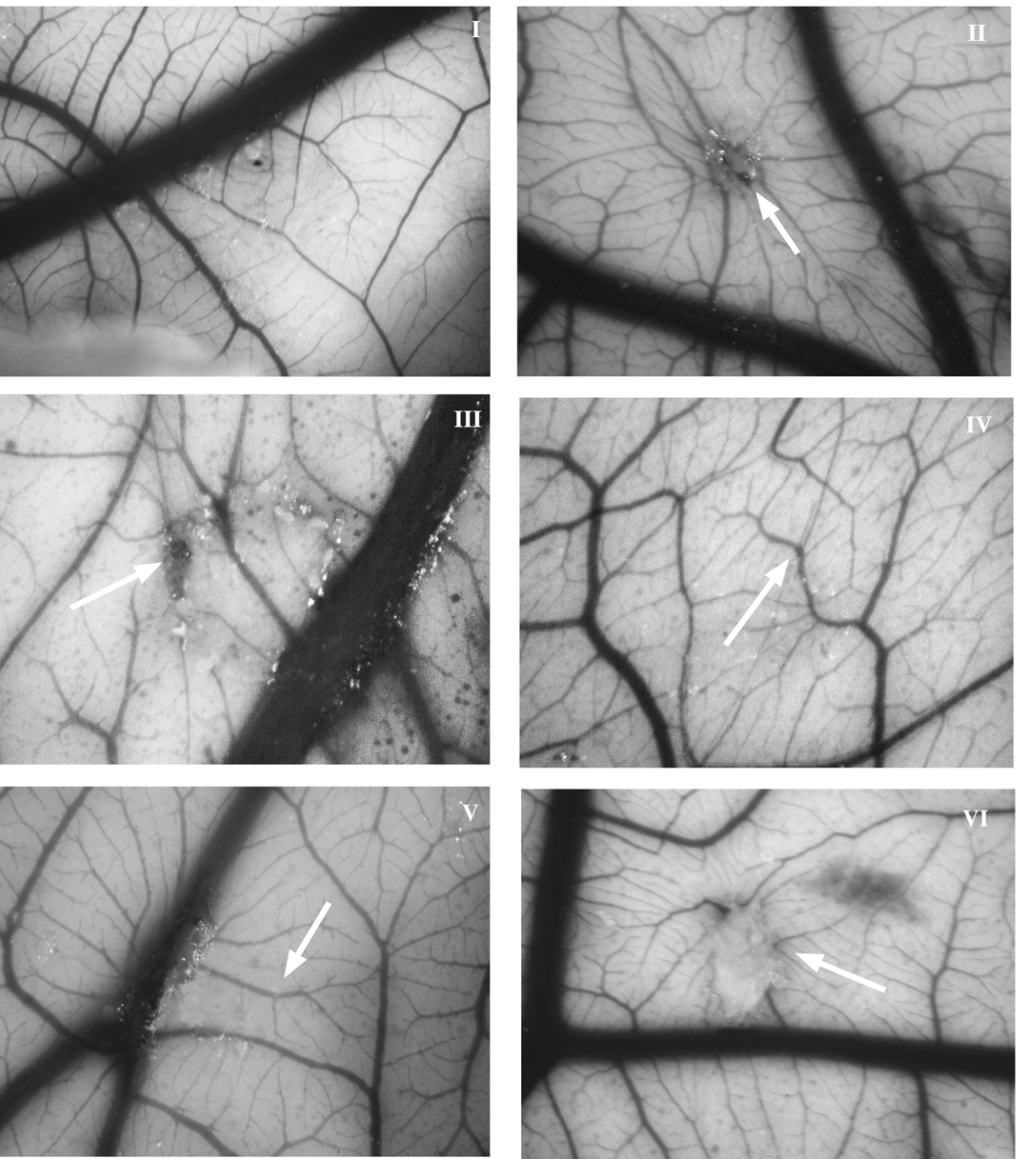
**Compounds 1 and 2 inhibit *in vivo *angiogenesis in the CAM assay (see below for method)**. (I) methylcellulose alone showed no evidence of angiogenesis or inflammation, (II) FGF-2 induced a network of new blood vesselsradiating from the site of application (arrow), (III) and (IV) compounds **1 **and **2 **(10 μg) alone again showed no inflammatory or angiogenic response at the site of application. (V) and (VI) When compounds **I **and **2 **(10 μg) were added together with FGF-2 there was complete inhibition of blood vessel formation. Representative photomicrographs are shown and the arrow indicates the point of application of the test compounds. Original magnification × 40.

Since the *in vitro *assays described above suggest inhibition of several steps of angiogenesis we next studied the interaction of compounds **1 **and **2 **with FGF-2 in an *in vivo *system, the CAM assay. There was no evidence of angiogenesis or inflammation with the control, methylcellulose, used for the addition of the test compounds (Figure 10i). After exposure to FGF-2 (25 ng) there was a significant increase in the formation of new blood vessels growing radially towards the stimulus (Figure 10ii) (m = 3, p < 0.001, n = 15). The determination of the degree of angiogenesis (m) is described below in the materials and methods section. After 6 days exposure to the test compounds (10 μg in each case) there was a 100% reduction in FGF-2-induced angiogenesis in the case of compound **1 **(Figure 10v; m = 0, p < 0.0001, n = 8) and 90% reduction with compound **2 **(Figure 10vi; m = 1, p = 0.0186, n = 5) with a notable inhibition in the formation of normal CAM blood vessels. The test compounds alone were not inflammatory or angiogenic (Figure 10iii and 10iv).

### Hydrophobicity of 1 and 2

The hydrophobicity descriptor (log P; a measure of the relative hydrophobicity) for compounds **1 **and **2 **were -0.07 and 0.76 respectively indicating that compound **1 **is approximately an order of magnitude more water soluble than **2 **but both compounds are very water soluble.

### The effect of compounds 1 and 2 on EGF and VEGF-induced angiogenesis

Compounds **1 **or **2 **had no significant inhibitory effect on EGF or VEGF-induced BAEC- or HDMEC growth or differentiation as measured in the angiogenesis assays described above (Table 1).

## Discussion and conclusion

We have demonstrated the ability of compound **1**, a plant-derived stilbene glycoside to inhibit multiple stages of angiogenesis in *in vivo *and *in vitro *assays with IC_50 _values in the range of 5.8 ± 0.18 to 48.90 ± 0.40 μM. Compound **1 **was equally effective against small and large vessel-derived ECs but compound **2 **was largely ineffective. Compound **1 **which contains an additional bulky L-rhamnose residue and is approximately 10 times more water soluble than compound **2 **was the more effective inhibitor. Since the compounds show approximately 90% structural homology, activity appears to be inversely related to hydrophobicity. We have shown using other plant-derived natural products that increased polarity correlates with effectiveness of growth factor inhibition. For example, the ability of sesterterpenes to inhibit FGF-2-induced angiogenesis [18] and cheiradone to inhibit VEGF [19] increased with polarity.

Stilbene glycosides and their derivatives are naturally occurring phytoalexins, widely distributed and with diverse activities. For example resveratrol, a stilbene glucoside inhibited the differentiation of human umbilical vein ECs into capillary-like structures at concentrations of 0.1–1 mM [20] and induced apoptosis in colon cancer cells [21]. Angiogenesis is an invasive process that requires degradation of the basement membrane, cell migration and removal of obstructing matrix proteins to allow space for the formation of the vessel lumen [22]. Matrix metalloproteinases (MMPs), in particular MMP-2 and -9 are the principal mediators of these events [23]. Stilbene glycosides are potent anti-oxidants with IC_50 _values in the μM range [24] and may suppress MMP expression, and therefore angiogenesis by decreasing oxidative stress, a known inducer of MMP-9. This may indirectly regulate angiogenesis [25]. However, we were able to show that the major effect of compound **1 **was in reducing FGF-2 induced angiogenesis. In the absence of growth factors, stilbene glycosides had little effect on EC proliferation and migration. However, compound **1 **bound to FGF-2 reducing its binding to its cognate receptors (FGFR-1 and -2) with IC_50 _values of 5.37 to 9.32 μM respectively. The effect was specific with no inhibition of EGF or VEGF activity and compound **1 **did not bind *in vitro *to the VEGF receptor. Angiogenesis is regulated by a balance between pro- and anti-angiogenic regulators. If the pro-angiogenic stimulators predominate ECs switch to an angiogenic phenotype. It appears that stilbene glycosides regulate angiogenesis by decreasing the activity of pro-angiogenic FGF-2. In addition compounds **1 **and **2 **showed activity in the *in vivo *CAM assay. The suppression of FGF-2 induced vascular networks was accomplished without inflammation or embryo death. *In vitro*, compound **1 **reduced the formation of vascular networks by BAECs and HUVECs on Matrigel beds and caused regression of established networks. Compound **1 **also reduced FGF-2 induced proliferation and migration of ECs. It is possible therefore, that compound **1 **may have a role in reducing excessive angiogenesis.

Activation of signal transduction pathways follows binding of growth factors to receptor tyrosine kinases and in the case of FGF-2 involves phosphorylation of ERK-1 and -2, culminating in the activation of the transcription factor c-jun and initiation of the early events of angiogenesis [26]. We were able to show that compound **1 **reduced levels of phospho-ERK-1 and -2 in FGF-2 stimulated ECs in a dose-response manner.

The few known natural product inhibitors of FGF-2 include two sesterterpenes, leucosesterterpene [18] and torilin [16] and the aporphine alkaloid sinomenine [27]. Both torilin and sinomenine show potential as anti-tumour agents, the latter is active against synovial carcinoma [27]. Since compound **1 **shows anti-angiogenic activity with similar IC_50 _values to sinomenine there is a possibility that it may have potential as an anti-tumour agent.

## Methods

### Angiogenic inhibitors

Two stilbene glycosides, *trans*-4',5'-dihydroxy-3-methoxystilbene-5-*O*-{α-L-rhamnopyranosyl-(1→2)- [α-L-rhamnopyranosyl-(1→6)}-β-D-glucopyranoside (compound **1**) and *trans*-4',5'-dihydroxy-3-methoxystilbene-5-*O*-[α-L-rhamnopyranosyl-(1→6)]-β-D-glucopyranoside (compound **2**) (Figure 1) were isolated from the medicinal plant *Boswellia papyriferia*. Their isolation and structural and chemical characterisation is described in detail elsewhere [17].

### Materials

Matrigel was obtained from Becton Dickinson, UK; FGF-2 and goat anti-active caspase-3 antibody from R&D Systems; VEGF_165 _from Apollo Cytokine Research, (Cambridge, UK), EGF, VEGFR-1 and -2, FGFR-1 and -2, anti-FGF-2 and anti-VEGF antibodies were obtained from Santa Cruz Biotechnology (Heidelberg, GDR). ABTS peroxidase substrate kit (Vector, UK), Transwell chamber system, culture plates and flasks (SLS, UK), anti-goat Alexa flour 488 conjugated green fluorescence dye and other chemical of commercial grade were purchased from Sigma (Poole, UK).

### Cell culture

Human dermal microvascular endothelial cells (HDMECs) and the appropriate medium were purchased from TCS Cellworks (Buckingham, UK) and were cultured and maintained according to the supplier's instructions. Bovine aortic endothelial cells (BAECs) were isolated and characterised as described previously [28]. They were routinely cultured in Dulbecco's modified Eagles medium (DMEM) in 5% CO_2 _at 37°C containing varying concentrations of foetal calf serum (FCS) as described above. All cells were used between passages six to nine.

### Cell proliferation studies

Cells were seeded in triplicate at a concentration of 6.0 × 10^5^/ml, in 2 ml of complete medium in 6-well plates. After attachment (24 h), medium was replaced with serum poor medium (SPM), containing 2.5% FCS in which the cells grew at a significantly reduced rate. Growth factors, FGF-2 (25 ng/ml), EGF (75 ng/ml) and VEGF (10 ng/ml) [29] and test compounds at different concentration were added and cells incubated for a further 72 h. Control wells were treated with 5 μl DMSO. Concentration ranges of test compounds and pre-incubation times were based on pilot studies. After 72 h, cells were washed in PBS, detached in 0.05%w/v trypsin in PBS, and counted on a Coulter counter (Coulter Electronics, Hialeah, FL) set to a threshold of 30 μm. Experiments were performed in triplicate and repeated at least twice.

### Chemotaxis assay

The effect of compounds **1 **and **2 **on cell migration was examined *in vitro *using a modified Boyden chamber system with 8.0 μm pore polycarbonate filter inserts (TSL, UK). The filter was coated overnight with 0.1%w/v gelatine, and air-dried. Cells (1 × 10^5^) were placed in the upper part of the filter and test compounds at different concentrations with and without growth factors were added to the lower part in SPM. Cells were incubated at 37°C for 6 h and the filter removed and the upper side containing non migrated cells wiped and rinsed. The filters were fixed (4% paraformaldehyde), and stained (Geimsa) and cell migration in duplicate wells was determined by counting cell numbers on the lower surface. Experiments were performed in triplicate and at least two times.

### Endothelial cell migration in wound healing

Cells (6 × 10^4^/ml) were added to a Thermanox cover slip in a 24-well plate in complete medium and incubated for 24–48 h. When confluent, the medium was replaced with DMEM containing 0.1% FCS and incubated for a further 48 h. Cover slips were washed (PBS, × 3), wounded with a sterile razor to produce a straight edged cut and washed in PBS to remove dislodged cells. Cover slips were added to a fresh 24-well plate in 0.1% FCS and incubated with FGF-2 (or other growth factors) and a range of concentrations of test compounds for 18 h. Under these conditions, there was negligible proliferation but measurable migration. Slides were fixed in ethanol (100%), stained with methylene blue and photographed. For each slide, 10 fields of view (2 mm × 1.45 mm) were counted at random. Each experiment was performed in triplicate and at least twice.

### Cell differentiation and invasion assays in Matrigel

Cells (1.0 × 10^6^/ml) were mixed with an equal volume of Matrigel (Becton -Dickinson, Oxford, UK; prepared according to the supplier's instructions) at 4°C. Aliquots (80 μl) were added to the wells of a 48-well plate and allowed to polymerise (1 h) then 500 μl of 15% FCS containing FGF-2 (25 ng/ml; or other growth factors), with or without test compounds was added. The cells were incubated for 24 h at 37°C then fixed in 4% paraformaldehyde (5 min), washed in cold ethanol and the plate air dried. Cells were stained with Geimsa (30 s), air dried and photographed. Ten random fields were selected and the number of closed tubes counted.

The procedure described above was repeated and wells having a uniform network of tubes were used to assess invasion into a second layer of Matrigel. Matrigel (100 μl), with and without the test compounds was added to the cells and allowed to polymerise (1 h at 37°C). FCS (15%) was added and the cells incubated for 24–72 h when tube growth into the upper layer was measured as described above.

Tube regression under the influence of the test compounds was also investigated. Cell suspensions were mixed with Matrigel supplemented with FGF-2 (25 ng/ml) in 15% FCS as described above. The gel was allowed to polymerise (1 h) and FCS (0.5 ml with FGF-2, (25 ng/ml) added and cells incubated for 24 h. Wells which had a uniform network of tubes (assessed with a Nikon inverted microscope) were treated with medium (0.5 ml, 15% FCS) containing test compounds. Plates were incubated for 24 h, fixed and stained as described above and tube formation counted. All experiments were performed in triplicate and at least two times.

### Binding assay

Competition between growth factors, their cell surface receptors and test compounds was assessed in an ELISA assay as described by [30]. A 96-well plate was coated overnight with 2 μg/ml of the soluble receptors (FGFR-1 or -2 and VEGFR-1 or -2) and blocked with 1% BSA in PBS containing 0.05% Tween-20. The test compound and FGF-2 or VEGF were pre-mixed for 2 h and added to the plate and incubated for 2 h. The plate was washed (× 3 with PBS-Tween-20) and incubated with anti-FGF-2 IgG (1:500 in PBS-Tween-20) or anti-VEGF IgG (!:250 in PBS-Tween-20) for 45 min, washed and incubated with horseradish peroxidase conjugated goat anti-IgG (Santa Cruz, 1:1000) for a further 45 min. After washing (× 3), ABTS peroxidase substrate was added and the absorbance read at 405 nm. IC_50 _values were calculated from the data using the EZ-Fit enzyme kinetic software (Perella Scientific Inc., Amherst, USA).

### Western blotting

The method is described in detail elsewhere [18]. In brief, cells were lysed in RIPA buffer, protein concentration determined using the Biorad assay, and approximately 20 μg aliquots separated by 12% SDS-PAGE, electroblotted onto nitrocellulose filters (Hoefer, San Francisco, Ca, USA), blocked overnight with 1% BSA in TBS-Tween and incubated for 4 h at RT with characterised antibodies to phosphor-ERK1/2 (AutogenBioclear, mouse monoclonal, 1:1000). α-Actin (Sigma, UK; 1:1000) was used as a loading control. Filters were washed in TBS-Tween and stained with antibodies to peroxidase-conjugated secondary antibody. Proteins were detected with the ECL system (Amersham, UK). Protein expression was estimated spectrophotometrically from band intensity. Results are semi-quantitative and given a numerical value compared to the weakest observed band assigned an arbitrary value of 1.0. All experiments were performed at least twice and representative examples are shown.

### Chick chorioallantoic assay

The angiogenic activity of test compounds was determined using the semi-quantitative chick chorioallantoic assay (CAM) as described previously [31]. To expose the CAM a window was created in the shells of 4 day-old chicken eggs. On day 8, a 2 mm^3 ^methycellulose pellet (5 μl of 1% sterile methylcellulose; 400 centipoise, Sigma UK) containing no additions (control), the test compound (10 μg) with and without FGF-2 (100 ng) were applied to the membrane. The resultant angiogenesis scored on day 14 as 0- negative; 0.5- change in vessel architecture; 1- partial spokewheel (1/3 of circumference exhibits directional angiogenesis); 2- spokewheel; 3 or greater- strong and fully spokewheel. This approach enabled calculation of an accumulated response in each group. To photograph the membrane, 2 cm^3 ^of a 50% emulsion of aqueous paraffin oil containing 2% Tween-80 was injected at the site of application and photographed using a Leitz dissecting microscope. Each experiment was performed five times and statistical significance was determined by the Mann-Whitney U test and the data is expressed as a median value (m).

### Toxicity

Stilbene glycoside toxicity was determined using the MTT and active caspase-3 assays. BAECs or HDMECs (7.5 × 10^3^) were seeded in a 96 well plate and incubated for 4 h to allow cell adhesion. The test compound or staurosporine (1 μM), an inducer of active caspase-3 and therefore of apoptosis was added to the wells. Control cells were treated with PBS and the plate was incubated at room temperature for 72 hours. MTT reagent (10 μl) was added followed by incubation at room temperature for 2–4 h. When a purple precipitate was visible, detergent reagent (100 μl) was added to the plates and incubated at room temperature for 2 h in the dark. Absorbance was measured at 570 nm using a microplate reader.

In the apoptosis assay, HDMECs or BAEC (4 × 10^4^/ml) in complete medium were added to the chambers slide and allowed to adhere for 24 h. The test compound (X μM) or staurosporine (1 μM) were added to all wells except control (PBS) and incubated for 24 hours. Wells treated with staurosporine were immediately washed (PBS) and fixed (4% paraformaldehyde) when cell morphology became round (2–4 h). After washing and fixing, cells were permeablized (0.1% Triton X-100; 10 min), washed (× 5~5 min each), air dried and blocked with 1% BSA in 1:50 TBS-Tween for 1 h at room temperature. Cells were incubated with goat anti-active caspase-3 (R&D system, UK; 1% BSA in TBS-Tween) for 1 h. The plates were incubated with anti-goat Alexa-Flour 488 conjugated green fluorescent dye for 1.5 h at room temperature. Ten random homogeneous fields were viewed, and photographed.

### Determination of the hydrophobicity of 1 and 2

The SdQSAR program [32] (Tripos, St Louis, Mo) was used to determine the octanol-water partition coefficient a measure of hydrophobicity.

### Statistical analysis

All data were expressed as mean ± SEM. Statistical analysis was performed by one way analysis of variance and a value of p < 0.05 was considered statistically significant.

## Authors' contributions

SH carried out the angiogenic assays. JG, MS and NA participated in the design of the study and JG prepared the manuscript. MIC and HN isolated and characterised the stilbene glycosides. DW performed the CAM assay. All authors read and approved the manuscript.
